# Signature Channels of Excitability no More: L-Type Channels in Immune Cells

**DOI:** 10.3389/fimmu.2015.00375

**Published:** 2015-07-23

**Authors:** Bennett Davenport, Yuan Li, Justin W. Heizer, Carsten Schmitz, Anne-Laure Perraud

**Affiliations:** ^1^Department of Biomedical Research, National Jewish Health, Denver, CO, USA; ^2^Department of Immunology and Microbiology, University of Colorado Denver, Denver, CO, USA

**Keywords:** calcium signaling, lymphocytes, myeloid lineage, Ca_V_ ion channels, L-type channels, DT40 B-cells, dihydropyridines

## Abstract

Although the concept of Ca^2+^ as a universal messenger is well established, it was assumed that the regulatory mechanisms of Ca^2+^-signaling were divided along the line of electric excitability. Recent advances in molecular biology and genomics have, however, provided evidence that non-excitable cells such as immunocytes also express a wide and diverse pool of ion channels that does not differ as significantly from that of excitable cells as originally assumed. Ion channels and transporters are involved in virtually all aspects of immune response regulation, from cell differentiation and development to activation, and effector functions such as migration, antibody-secretion, phagosomal maturation, or vesicular delivery of bactericidal agents. This comprises TRP channel family members, voltage- and Ca^2+^-gated K^+^- and Na^+^-channels, as well as unexpectedly, components of the Ca_V_1-subfamily of voltage-gated L-type Ca^2+^-channels, originally thought to be signature molecules of excitability. This article provides an overview of recent observations made in the field of Ca_V_1 L-type channel function in the immune context, as well as presents results we obtained studying these channels in B-lymphocytes.

## Introduction

Virtually all biological processes, including immune responses, require at some stage the carefully orchestrated elevation of cytoplasmic Ca^2+^ by various molecular mechanisms, which provide crucial cellular signals. When it comes to molecular components of ion flow regulation, it was commonly assumed that excitable cells such as neurons or cardiac muscle cells possess a panoply of unique ion channels and transporters representing the molecular tools of electrical excitability. In contrast, non-excitable cells such as immunocytes were thought to mainly, if not even in some cases exclusively, rely on store-operated Ca^2+^-entry (SOCE) to generate Ca^2+^-signals. This pathway, also called capacitative Ca^2+^-entry (1), is triggered by the activation of cell surface receptors and subsequent activation of phospholipases that generate the soluble second messenger IP_3_ (Inositol 3-Phosphate). Through binding to IP_3_-receptors at the surface of the endoplasmic reticulum (ER), IP_3_ elicits the depletion of the ER Ca^2+^-stores resulting in a first phase of Ca^2+^-elevation. In response to this store-depletion event, Ca^2+^-permeable channels at the plasma membrane get in turn activated, leading to Ca^2+^-entry from the extracellular space and to a sustained phase of cytosolic Ca^2+^-elevation and replenishment of the stores. In recent years, the molecular identity of the proteins underlying SOCE has been unveiled. The STIM proteins were identified as the ER Ca^2+^-sensors, which in response to ER store depletion interact with, and open the pore of the ORAI ion channels in the plasma membrane (2–4). Extensive functional studies in various populations of immune cells have become available. Unexpectedly, genetic deletion of these molecular components of store-operated Ca^2+^-entry has revealed that immune cell development and activation is not as reliant on this Ca^2+^-entry pathway as originally hypothesized. For example, although Ca^2+^-signals are known to be generated during development and selection of thymocytes, neither humans with genetic deficiencies in ORAI and STIM proteins, nor genetically engineered mice lacking SOCE show impaired T-cell development and selection (5–7). The same is true of B-cell development as no anomalies were found in B-cell populations in the bone marrow and secondary lymphoid organs of patients with mutations in the *Orai1* or *Stim1* genes (6, 7), or in mice with defects in these same molecules or STIM2 (5, 8), despite deficient B-cell receptor (BCR)-mediated Ca^2+^-signaling. On the other hand, T-Lymphocyte activation is SOCE-dependent, as illustrated by STIM1/ORAI1 deficient humans who exhibit lymphoproliferative defects and severe combined immunodeficiency (SCID), a phenotype consistent with SOCE-deficient mouse models, although murine STIM/ORAI proteins show a higher level of functional redundancy (9).

Following the crucial characterization of STIM and ORAI and the availability of expression datasets in various immune cell populations, it has become increasingly clear that beyond SOCE, non-excitable immune cells possess a large and diverse pool of ion channels involved in all aspects of immune response regulation. This includes numerous members of the TRP channel family of cationic channels, voltage- and Ca^2+^-gated K^+^-channels, and also, surprisingly, voltage-gated Sodium channels (10), and components of the Ca_V_1 subfamily of L-type voltage-gated Ca^2+^-channels (VGCC), originally thought to be signature molecules of excitability (11–14).

In excitable cells, voltage-dependent Ca^2+^-entry has been extensively characterized biophysically and pharmacologically. These currents were subdivided into several subclasses based on these electrophysiological and pharmacological properties (15, 16). Molecules mediating “Long-lasting” L-type currents are commonly described as high voltage-activated channels with comparatively slow activation and rapid deactivation. Another important hallmark of L-type channels in the excitable context is the strong Ca^2+^-dependence of their inactivation, and their inhibition by 1,4-dihydropyridines (DHPs). L-type Ca^2+^-channels are often mentioned as signature channels of excitability since they couple excitation to contraction in skeletal, cardiac, and smooth muscle cells. They are also present in neurons and endocrine cells where they participate in a wide range of biological processes from cell death to transcriptional regulation or hormone secretion. Although immune cells are not known to undergo massive membrane depolarization, and lack the typical voltage-activated Ca^2+^-entry linked to L-type channels in the excitable context, there is mounting evidence that pore-forming L-type VGCC α1 subunits, as well as accessory β-subunits, are functionally expressed in various types of immunocytes, including B- and T-lymphocytes, but also in cells of the myeloid lineage (12–14, 17, 18). L-type channel blockers are commonly used to treat cardiovascular conditions such as high blood pressure. Understanding the role of these channels in the context of immunity and inflammation is therefore also relevant therapeutically. Before reviewing the current knowledge about the presence and potential involvement of L-type channels in the immune system, a brief overview of their structure, regulation, and biology will be given.

## Topology, Nomenclature, and Regulation of Voltage-Gated Ca^2**+**^ Channels

**Figure 1:**
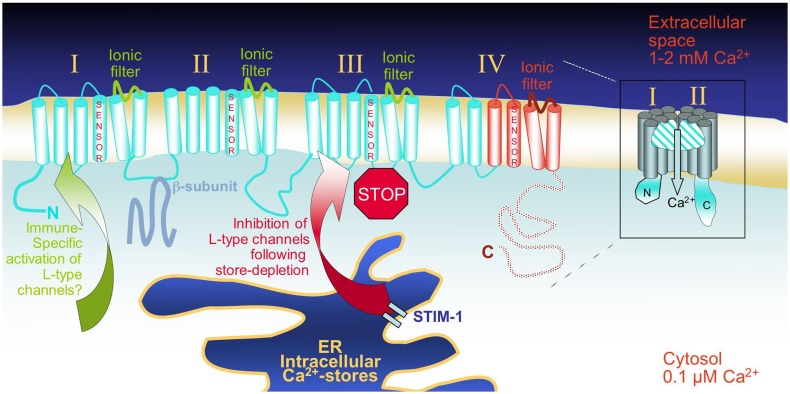
**Schematic representation of the overall predicted topology and of the pore forming Ca_V_α1 subunits: The 24 transmembrane (TM) regions of a typical Ca_V_1 pore-forming unit are depicted in a linear fashion**. In red are the C-terminal protein segments that were found to be affected by alternative splicing in T-cells, and might as a result be partly lacking in these cells. On the right, one channel is represented as a “half-pore” cross section through the plasma membrane. Ca_V_α1 subunits are assumed to show the same fold as other cationic channel families, which is characterized by the formation of a pore through four groups (I–IV) of six TM domains that line the ionic selectivity filter. The voltage-gated sensor that is essential for gating in excitable cells is located on TM4 of each group. L-type channels are currently discussed as “store-inhibited channels,” representing a novel mechanism of Ca^2+^-signal coordination – upon depletion of the ER Ca^2+^-stores, the ER Ca^2+^-sensing STIM proteins activate store-operated Orai-channels, but at the same time, repress L-type channels. The mode of activation of L-type channel family members in various immune cell types remains to be determined.

The α1 pore-forming subunits of VGCCs are predicted to contain a total of 24 transmembrane (TM) spans arranged in four groups of six spans where the fourth one functions as a voltage sensor, and the loop between the fifth and sixth span is part of the channel’s ionic selectivity filter (Figure 1). This overall topology is common to several other families of cationic channels, such as TRP (transient receptor potential), K_v_ (voltage-gated K^+^) or CNG (cyclic nucleotide-gated) channels, that all harbor the same TM architecture. One main difference to VGCCs is, however, that in all these other channels the four groups of six TM spans are expressed as single independent entities that tetramerize to form a complete pore, allowing for the heteromultimerization of several members of the same channel family. Although the pore-forming subunit of VGCCs is in one continuous polypeptide chain, VGCCs are also multi-subunit complexes where the pore-forming α1 subunit interacts with regulatory/auxiliary subunits designated β, α2/δ, and γ, which are playing an essential role in regulating trafficking and assembly, but also in shaping channel activity features such as kinetics of activation or inactivation. Ten distinct genes subdivided into three phylogenetic subfamilies have been found to encode α1 subunits in mammals. In an effort to reorganize the nomenclature, VGCCs have been renamed Ca_V_ (for voltage-gated Ca^2+^), followed by the subfamily number (1–3), and the particular member number. L-type currents are mediated by four different α1 subunits, now called Ca_V_1.1 to 1.4 (formerly α1S, α1C, α1D, and α1F, gene names are *cacna1s*, *cacna1c*, *cacna1d*, and *cacna1f*, respectively) (19). Multiple splice variants from all subunits listed above further contribute to the amazing molecular plasticity of these channels [reviewed in Ref. (16, 20)]. It was, for example, described that for the gene encoding human Ca_V_1.2, at least 20 from the 50 exons can be subjected to splicing (16). In T-lymphocytes, two splice variants of Ca_V_1.4 (α1F) have been found, one lacking major parts of the fourth group of TM spans (IVS3–S6), including the voltage sensor and the pore loop, and another variant lacking part of the extracellular loop connecting IVS3 with S4 (21) (Figure 1). As a result of the deletion of exon 37, both these T-cell-specific splice variants exhibit a novel and shorter C-terminal end that surprisingly shows significant homology to Ca_V_1.1. As might have been expected because of the lack of typical voltage-gated Ca^2+^-entry, it therefore appears that T-cell-specific versions of Ca_V_1 channels exist, although they have not yet been functionally characterized. A similar Ca_V_1.3 variant had been described several years prior in a rat hepatoma cell line, and was also assumed to be voltage-insensitive, and thus gated via different mechanisms than in excitable cells (22). In addition to the extensive mRNA splicing, multiple reports have documented the proteolytic cleavage of the C-terminal portions of Ca_V_1.2 (α1C) and Ca_V_1.3 (α1D), resulting in truncated channels with altered biophysical properties (23–25). Recent studies have shown that the Ca_V_1.2 C-terminal fragment translocates in a Ca^2+^-dependent manner into the nucleus, where it acts as a positive and negative regulator of transcription (26, 27). This finding further emphasizes the complex and multilayered physiological functions of these molecules. Importantly, the predicted topology shown in Figure 1 results in both protein termini being cytoplasmic, provided the channel is inserted into the plasma membrane. The gating of these channels is regulated at multiple levels, and often involves the interaction of their cytoplasmic regions with gating agents, as well as with other modulatory proteins that adjust the extent and kinetics of channel activation to cellular needs. Several molecules can bind to the same region of a given channel, like calmodulin (CaM), CaBP1, and CaMKII, which all interact with the N-terminal tail of Ca_V_1.2, potentially resulting in a competition between these proteins to regulate channel kinetics (28). One extensively studied example is the modulation of voltage-gated Ca^2+^ channels by the Ca^2+^-sensor CaM [reviewed in Ref. (29)].

## Store-Operated vs. L-Type Channel-Mediated Ca^2**+**^-Entry: Unexpected Reciprocity

An unforeseen consequence of identifying the molecular components of store-operated Ca^2+^-entry – the ER Ca^2+^ sensor proteins STIM1/2, and the STIM-activated Orai (also CRACM1) plasma membrane channels – has been the realization that VGCCs are “store-inhibited” channels (30). As several studies have documented, there appears to be a reciprocal relationship between store-operated and VGCC-mediated Ca^2+^-entry pathways ensuring a coordinated activation pattern of these two major Ca^2+^-signaling pathways. Two simultaneous reports in 2010 described how the ER Ca^2+^-sensor STIM1 not only activates Orai-channels, but also suppresses the activation of Ca_V_1.2 (31, 32). As a similar relationship was recently discovered between STIM1 and T-type Ca^2+^-channels (33), this reciprocity might represent a common mode of Ca^2+^-signal coordination. Moreover, beyond IP_3_ receptors, STIM proteins have also been shown to colocalize with the intracellular Ca^2+^ release ryanodine receptors (RyR) in T-cells, indicating the functional coupling of RyR and SOCE (34). A tight relationship also exists between the SOCE machinery and the SERCA pumps that are responsible for refilling the ER stores (35). Thus, STIM is emerging as a molecular hub for cellular Ca^2+^ homeostasis regulation. This could have important implications when considering the physiological and potentially pathological consequences of STIM function alterations.

## Evidence for L-Type Channels in the Immune Context

The presence of L-type channels in immune cells is mostly discussed in lymphocytes, but several studies also describe their occurrence in cells of the myleoid lineage. As mentioned before, the DHP sensitivity of L-type channels has been extensively used to assess the role of these channels under various circumstances and in diverse cell types. Since these drugs have been applied therapeutically already for decades and are “valuable and widely used agents in the management of essential hypertension and angina” (36), researchers early on have investigated the possible effect of these therapeutics on lymphocytes, and found that Ca^2+^-flux and cell proliferation in response to activation are diminished in lymphocytes exposed to these compounds. The potential immunosuppressive action of DHPs, in particular at the comparatively low concentrations used clinically, remains however uncertain. A possible synergy between DHPs and cyclosporine A, a combination frequently used in transplantation patients, has been described (37).

An important clue to the potential role of L-type channels in immunity comes from patients with rare genetic diseases affecting Ca_V_1 ion channels, and who also suffer from immune impairments. Because cardiac issues are usually severe and lead to the most acute and life-threatening health crises in these children, the known immune defects they present have only been characterized superficially. Timothy syndrome (TS) is a complex disorder caused by point mutations in the Ca_V_1.2 gene. The mutated channels were found to generate sustained inward Ca^2+^-currents originating from an almost complete loss of voltage-dependent channel inactivation (38). Severe infections, particularly bronchial and sinus infections, show a higher incidence and severity in individuals with TS, suggesting a significant role for Ca_V_1.2 in mounting a potent immune response in humans (39). In the following, an overview of the current knowledge about L-type channel function in major immune cell types will be given.

## L-Type Channels in T-Lymphocytes

Most of our knowledge about the involvement of Ca_V_ channels in the immune context was acquired in T-lymphocytes. As several excellent contributions in a recent special issue of Frontiers Immunology entitled “The Regulation of Calcium Homeostasis in T Lymphocytes” have provided in-depth reports about L-type channels in T-lymphocytes (13, 14, 40), we will only give a succinct review of this topic.

Although earlier reports had suggested the presence of members of the L-type family of ion channels in T-cells (22), it is mostly over the past decade that this unexpected finding has been more closely investigated. The group from Wilfred Jefferies published the first detailed evidence of Ca_V_ involvement in human T-cells by showing expression of *cacna1f* (Ca_V_1.4) in Jurkat T-cells in addition to primary peripheral blood CD4 and CD8 T-cells (11). The function of *cacna1f* expression was confirmed through a series of experiments utilizing L-type-specific DHP agonist and antagonist. These studies revealed that pharmacological manipulation of Ca_V_ channels in T-cells modulated T-cell receptor (TCR)-dependent Ca^2+^ flux, influenced phosphorylation and translocation of key TCR signaling pathways, regulated production of the proinflammatory cytokine IL-2 and upregulation of the IL-2R (CD25), and blunted TCR-induced proliferation. It has since been shown that both CD4^+^ and CD8^+^ T-cells express unique patterns of L-type Ca_V_ channels, dependent upon their lineage differentiation and activation status (13).

Useful insights have been gained from studies conducted in mouse models with alterations in genes encoding Ca_V_-channel subunits (41–43). Mice deficient in the cytoplasmic β-subunits β3 (knock-out), or β4 (spontaneous mutation), show normal intrinsic T-cell development – previously described anomalies in thymus development in the β4 mutant (44) are likely due to the onset of neuropathy exhibited in these mice at 2 weeks of age (41). It was further concluded through bone marrow chimera experiments that the β4 mutant contained a normal assortment of peripheral CD4 and CD8 T-cells, and a preserved naïve T-cell phenotype (CD44^lo^CD62L^hi^, CD25^−^CD69^−^). In contrast, β3-deficiency resulted in a dramatic reduction of splenic CD8^+^ T-cells, of which a significant portion presented with an activated phenotype (CD44^hi^CD62L^lo^) (42). TCR-mediated Ca^2+^ responses in CD4^+^ T-cells are diminished in both Ca_V_β3- and Ca_V_β4-deficient mice, directly contributing to impaired nuclear translocation of the Ca^2+^-sensitive transcription factor NFAT, leading to reduced production of the proinflammatory cytokines IFNγ and IL-4 (41, 42). Although CD4^+^ and CD8^+^ T-cells express unique profiles of β subunits (β4/β3 and β2/β3, respectively), it has yet to be clarified how these patterns directly regulate T-cell biology. Auxiliary subunits of Ca_V_ channels come in many different variants, increasing the molecular diversity of these channels. Functionally, auxiliary subunits enhance membrane expression of the channels, influence current properties, and shape the composition of signaling complexes associated with the channels. As subunit interactions of L-type channels are highly promiscuous, cell-specific expression patterns are crucial to define the type of channel complex being functionally expressed (45). Thus, it is plausible that the unique characteristics of each β subunit (subcellular localization coordination, phosphorylation status, complex formation with other adaptor/signaling molecules) could be an additional layer of T-cell regulation (42, 45).

Interestingly, CD8^+^ T-cells show a state-dependent expression pattern for Ca_V_1 channels. Although the CD8^+^ T-cell population as a whole expresses mRNA transcripts for all four members of the Ca_V_1 family, Ca_V_1.1 and Ca_V_1.4 exhibit differential regulation. Whereas Ca_V_1.4 protein is found in naïve but not in activated CD8^+^ T-cells, Ca_V_1.1 expression is the opposite during TCR stimulation, showing prominent expression in activated CD8^+^ T-cells, but not naïve. In addition, only the β3 regulatory subunit was shown to be prominently expressed in CD8^+^ T-cells (42). In this same study, utilizing β3-deficient mice, it was observed that peripheral naïve CD8^+^ T-cell homeostasis was dramatically altered. In the absence of β3, although thymic T-cell development was not perturbed, peripheral CD8^+^ T-cell numbers were greatly diminished. It was shown that CD8^+^ T-cells in β3^−/−^ mice exhibit a skewed activated phenotype (CD44^hi^CD62L^lo^) in the absence of any simulation/immunization, as well as increased activation-induced cell death, and impaired TCR-induced Ca^2+^ flux, proliferation, and effector cell function (as evident by lack of proinflammatory IFNγ, TNFα, IL-2 and granzyme B production).

The CD8^+^ T-cell phenotype and functional capacity in β3^−/−^ mice is partly reminiscent of exhausted T-cells in chronic viral infection as shown by the activated phenotype, impaired proliferative capacity and diminished cytokine production, although the cardinal marker for exhaustion PD-1 is not upregulated in β3^−/−^ CD8^+^ T-cells (42, 43, 46). In depth analysis of the molecular signatures of exhausted antiviral CD8^+^ T-cells revealed, among other things, an absence in NFAT nuclear translocation (46, 47). In addition to the absence of nuclear NFAT in exhausted CD8^+^ T-cells, alteration in the balance of NFAT and its binding partner AP-1 has dramatic effects on the targeted transcriptional program (48). When NFAT fails to complex with AP-1, the genes targeted are associated with anergy, tolerance, and exhaustion (48–50). Although the phenotypic and functional discrepancies observed in CD8^+^ T-cells in β3^−/−^ mice do not completely mirror those of exhausted antiviral CD8^+^ T-cells, it is convincing to predict that the culmination of TCR-mediated Ca^2+^ flux and coordinated regulation of NFAT nuclear translocation mediated by the Ca_V_1 β3 subunit has the potential to regulate the onset of CD8^+^ T-cell exhaustion.

It was also shown that the absence of β3 regulatory subunit resulted in a complete loss of cellular Ca_V_1.4 protein. The authors concluded from their observations that the Ca_V_1.4-β3 complex in naïve CD8^+^ T-cells contributes to antigen independent, MHC-triggered Ca^2+^ responses that are required for tonic signaling and the survival of these cells. This finding was confirmed and further expanded when the phenotype of mice constitutively lacking Ca_V_1.4 was characterized. Recapitulating previous investigations, these studies demonstrated that Ca_V_1.4 (*cacna1f)* is needed for the survival of peripheral naïve CD4 and CD8 T-cells. They further showed that Ca_V_1.4 deficiency significantly reduced both CD4^+^ and CD8^+^ antigen-specific T effector cell responses. When challenged with ovalbumin-expressing *Listeria*, *cacna1f*^−/−^ mice exhibited a significantly reduced expansion in antigen-specific CD4^+^ and CD8^+^ T effector cells, in addition to impaired CD8^+^ T-cell-mediated cytotoxicity (43).

Expression analyses of Ca_V_1 pore-forming subunits in different subsets of CD4^+^ Th cells have shown that Ca_V_1.2 and 1.3 are a hallmark of Th2 cells where they promote cytokine production (51, 52). Administration by airway inhalation of a combination of Ca_V_1.2 and Ca_V_1.3 antisense oligonucleotides provided protection against the development of Th2-dependent airway inflammation and hyperreactivity in mice (53), pointing at the therapeutic potential of airway-targeted inhibition of Ca_V_-channels for the treatment of asthma. In humans, gene expression analyses in circulating blood cells revealed expression of Ca_V_1.2, 1.3, and 1.4, as well as of accessory subunits required to form functional Ca_V_ channels (54). Comparably to mice, Ca_V_1.4 was detected in Th1 and Th17 cells, whereas Ca_V_1.2 is selectively expressed in Th2 cells. Although Ca_V_1.4 was down-regulated following TCR-stimulation of Th1 cells, this was not the case for Ca_V_1.2 in Th2 cells. Accordingly, pharmacological inhibition of TCR-mediated cytokine production was specifically reduced in Th2, but not in Th1, cells (54).

The differential expression profile of expressed Ca_V_ subunits in T lymphocytes is therefore expected to be an important factor in generating the diversity of Ca^2+^ signals required for T-cell development, homeostasis, and effector functions.

## L-Type Channels in B-Lymphocytes

The generation of Ca^2+^-signals in B-lymphocytes appears to be diversified and not as dependent upon STIM/Orai-mediated store-operated Ca^2+^-entry as originally anticipated (55). In mice lacking both ER Ca^2+^-sensor proteins Stim1 and 2 selectively in B-cells (Mb1-Cre-mediated deletion), B-cell development was normal, although SOCE was shown to be largely deficient. Antibody production, as well as LPS- and anti CD40-dependent B-cell proliferation, are also intact in these mice. However, BCR-mediated proliferation is strongly diminished, and B-cell-derived production of the anti-inflammatory cytokine IL-10 is severely reduced as a result of defective activation of the Ca^2+^-regulated transcription factor NFAT, leading to enhanced Th1-driven experimental autoimmune encephalomyelitis (EAE) (8).

Because many Ca^2+^-regulated B-cell functions remain intact in the absence of functional SOCE, it suggests that other Ca^2+^-signaling mechanisms must exist in these cells. Comparatively little is known about the role of L-type channels in B-lymphocytes. Several pharmacological studies have demonstrated that blocking L-type channels in human B-cells reduces BCR-induced Ca^2+^-entry (17, 56). In freshly isolated rat B-cells, an antibody against Ca_V_1.3 was found to block IgD-mediated Ca^2+^-responses (56). In this same study, it was confirmed that depolarization does not result in a Ca^2+^-response in B-lymphocytes, however, a cGMP-dependent protein kinase involvement was discovered. In the human L3055 B-cell line, gene and protein expression of Ca_V_1.2α1 and β1 was demonstrated, whereby the Ca_V_1.2α1 version expressed in these cells appeared to be non-voltage-gated and truncated in comparison to the version present in cardiac tissues (17). In this same study, an antibody raised against the extracellular region of Ca_V_1.2 was found to trigger a Ca^2+^-flux, indicating functional expression of this channel at the plasma membrane of L3055 B-cells. A prior report about a Ca_V_1.2α1 version expressed in murine erythroleukemia cells document the occurrence of putatively voltage-insensitive versions of the Ca_V_ channels expressed in non-excitable cells. This channel can be blocked by nifedipine, which partially inhibits differentiation of these cells. This Ca_V_1.2 variant lacks the first four segments of domain I of the pore-forming subunit, and does not produce measurable currents when expressed by itself in Xenopus oocytes (57). Therefore, the gating mechanism of this Ca_V_1.2 variant remains unknown. A study performed on subsets of sorted splenic mouse B-cells mentions that BCR-mediated Ca^2+^-mobilization in transitional T1 B-cells is more sensitive to the L-type channel blocker verapamil than that in T2 cells (58). From our review of the literature, there is currently no answer to the question under which circumstances and in which particular subsets of B-cells particular L-type channel subunits are expressed in vivo.

### Studying L-type channel function in DT40 B-cells

#### Gene and Protein Expression of Ca_V_1 Subunits in DT40 Cells

To further address the potential role of L-type Ca^2+^ channels in B-lymphocytes, we chose to utilize the chicken DT40 B-cell line that allows for the comparatively easy genomic disruption of genes of interest due to an unusually high rate of homologous recombination (59). DT40s have been shown to express an IgM isotype BCR at the cell surface, and BCR-stimulation by anti IgM leads to PLCγ2 activation and subsequent cytosolic [Ca^2+^] elevation. Signaling components of the avian BCR-pathway show very high conservation to their mammalian counterparts, and the DT40 system has been extensively used to characterize this pathway [reviewed in Ref. (60)].

**Figure 2:**
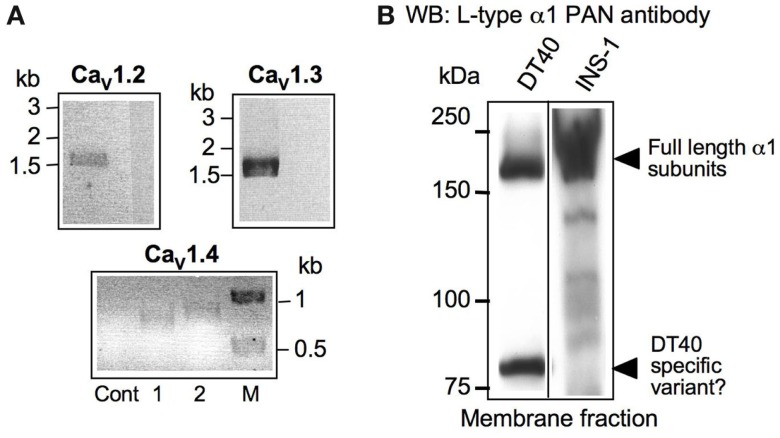
**Gene and Protein expression of Ca_V_1α1 family members in DT40 B-cells**. **(A)** RT-PCR using primer combinations specific for the indicated chicken version of Ca_V_1.2 (1.5 kb,), 1.3 (1.6 kb), and 1.4 (656 and 761 bp, lanes 1 and 2, respectively). M = marker. All PCR-fragments have been cloned and sequenced for verification. The negative control lanes (Cont) are reactions containing RNA as template. **(B)** Western blot from DT40 membrane fraction lysates developed using L-type α1 PAN antibody. As a positive control, the excitable pancreatic rat beta cell line INS-1 was used.

We first aimed at defining the expression pattern of individual pore-forming subunits of the Ca_V_1 L-type family of channels in DT40 wild-type cells. After identifying in the NCBI database the chicken homologs of the mammalian Ca_V_1.1, 1.2, 1.3, and 1.4, we performed RT-PCR experiments. We found all Ca_V_1 genes except Ca_V_1.1 to be expressed in DT40 cells (Figure 2A). The amplified ~1.5 kb from the 3′ end of Ca_V_1.2 and 1.3 DT40 cDNA were cloned and sequenced, and are encoding the complete cytoplasmic C-terminal domains of these two channels. In order to confirm that also proteins from these channels can be detected in DT40s, we conducted simple cell fractionation experiments, and used the excitable INS-1 rat pancreatic beta-cell line as a positive control. To visualize the channels, we used a PAN-antibody recognizing an epitope present in all L-type α1 pore-forming subunits (aa1506-1524 of rat Ca_V_1.1), which is conserved in the chicken version as well. In the DT40 cell membrane fraction, a band of the same size as a strong signal obtained in the INS-1 cells (~200 kDa) was detected, indicating that full-length α-subunits are expressed in DT40 B-cells (Figure 2B). Lower molecular weight bands were also seen, suggesting degradation or shorter splice variants. As these results were obtained using a PAN anti-Ca_V_1-antibody, we cannot conclude which combination of the three subunits whose genes are expressed, Ca_V_1.2, 1.3, or 1.4, is present at protein level. Because individual antibodies against each of the subunits are recognizing epitopes that are not well conserved in chicken, this question could not be further addressed using existing reagents.

#### Effect of L-Type Inhibitor on BCR-Mediated Ca^2**+**^-Signals in DT40s

**Figure 3:**
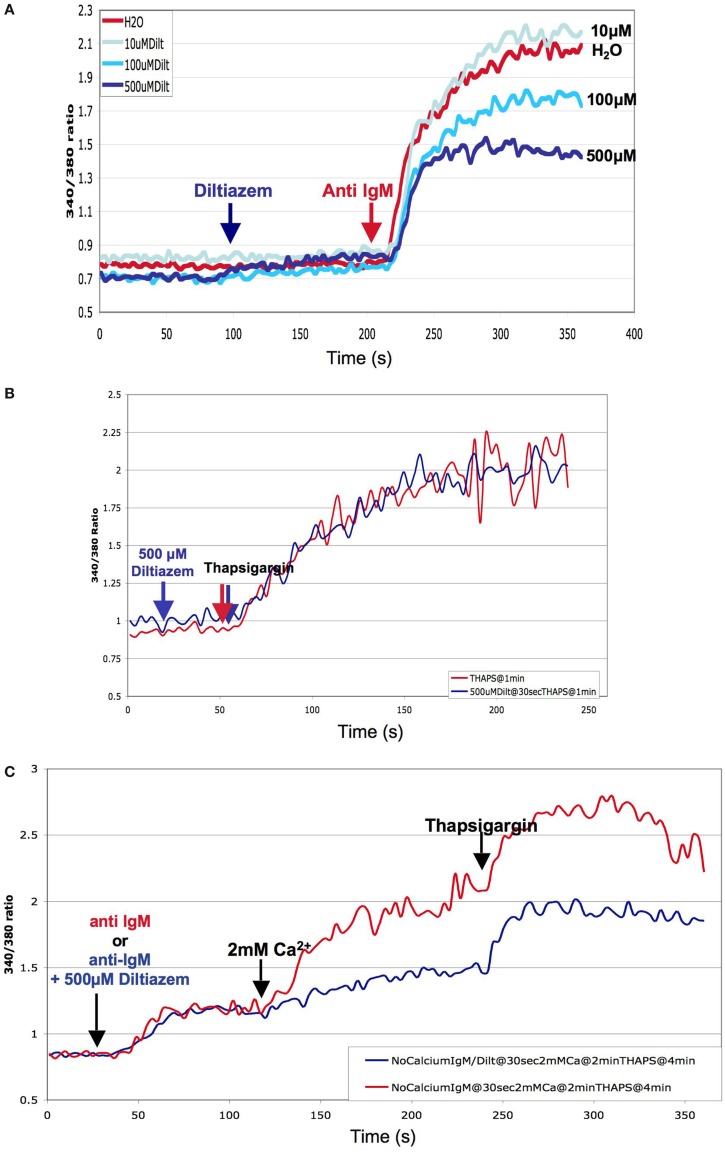
**Pharmacological inhibition of BCR-mediated Ca^2+^-response by the L-type channel blocker Diltiazem: DT40 cells were loaded with the fluorescent Ca^2+^-dye Fura-2 and the cells stimulated either through the addition of chicken anti-IgM (BCR-ligation) or thapsigargin (pharmacological store-depletion) into the cuvette**. The measurements were performed using a spectrofluorometer (Photon Technology International) at 37°C. **(A)** Dose dependence of the inhibitory effect of diltiazem on BCR-mediated cytosolic Ca^2+^-mobilization. **(B)** Effect of diltiazem on the store-operated Ca^2+^-response elicited by thapsigargin in DT40 cells **(C)** Diltiazem inhibition of the influx of extracellular Ca^2+^ following BCR-ligation. DT40 cells were loaded with the fluorescent Ca^2+^-dye Fura-2 in Ca^2+^-free buffer, and the cells stimulated through the sequential addition of chick anti IgM, 2 mM Ca^2+^, and thapsigargin into the cuvette.

As mentioned previously, several pharmacological studies using inhibitors of L-type channels such as nifedipine or diltiazem have shown a decrease of the Ca^2+^-response following receptor ligation in lymphocytes. We confirmed this finding in wild-type DT40 cells by showing a dose-dependent diltiazem-mediated ­inhibition of the Ca^2+^-signal following BCR-stimulation (Figure 3A). However, diltiazem had no effect on the pharmacological depletion of intracellular ER stores and subsequent activation of store-operated Ca^2+^-entry using the SERCA-pump inhibitor thapsigargin, suggesting that diltiazem targets a BCR-specific event that is not triggered by thapsigargin (Figure 3B). The anti-IgM-mediated depletion of the Ca^2+^-stores in the absence of extracellular Ca^2+^ was not inhibited by diltiazem (Figure 3C). Solely the second phase of Ca^2+^-elevation requiring entry from the extracellular space was affected, and the amplitude of Ca^2+^-elevation following exposure to thapsigargin was again unchanged by the addition of diltiazem. Noticeably, a similar pattern was observed in murine CD4^+^ T-cells lacking the Ca_V_β3-subunit required for proper trafficking of the Ca_V_1 subunits; a decrease in the amplitude of the Ca^2+^-response was only observed after cross-linking of the TCR, but not following the application of thapsigargin (41). In this context, it is interesting that a recent study characterizing Ca_V_1.4-deficient (*Cacna1f*^−/−^) mice has shown that whereas naïve CD44^lo^ CD4^+^ T-cells show a Ca_V_1.4 dependence for both their TCR- and thapsigargin-mediated Ca^2+^-responses, more mature CD44^hi^ CD4^+^ T-cells exhibit a pattern similar to the diltiazem effect in DT40 B-cells with only their TCR-mediated, but not their thapsigargin-induced Ca^2+^-response relying on Ca_V_1.4 (43). The molecular basis for this differential Ca_V_-dependence of ­pharmacological store-depletion vs. immunoreceptor-mediated Ca^2+^-signals in certain immune cell subsets remains to be elucidated.

Because DHPs need to be applied in the higher μM range to elicit an effect on the Ca^2+^-response of immunocytes, their specificity under these conditions has been questioned since an inhibitory effect on other channels such as K^+^ channels (K_V_ and K_Ca_) was shown in some studies. However, as discussed by Kotturi et al. (11), in these cases the reported effects were not consistent with the observations made in immune cells. It is therefore very probable that the Ca^2+^-entry pathway inhibited by diltiazem in DT40 cells originates from Ca_V_ variants that appear to be less sensitive toward this compound than their counterparts expressed in the excitable context.

#### Inducible Deletion of Ca_V_1.3α1 in DT40 B-Cells

**Figure 4:**
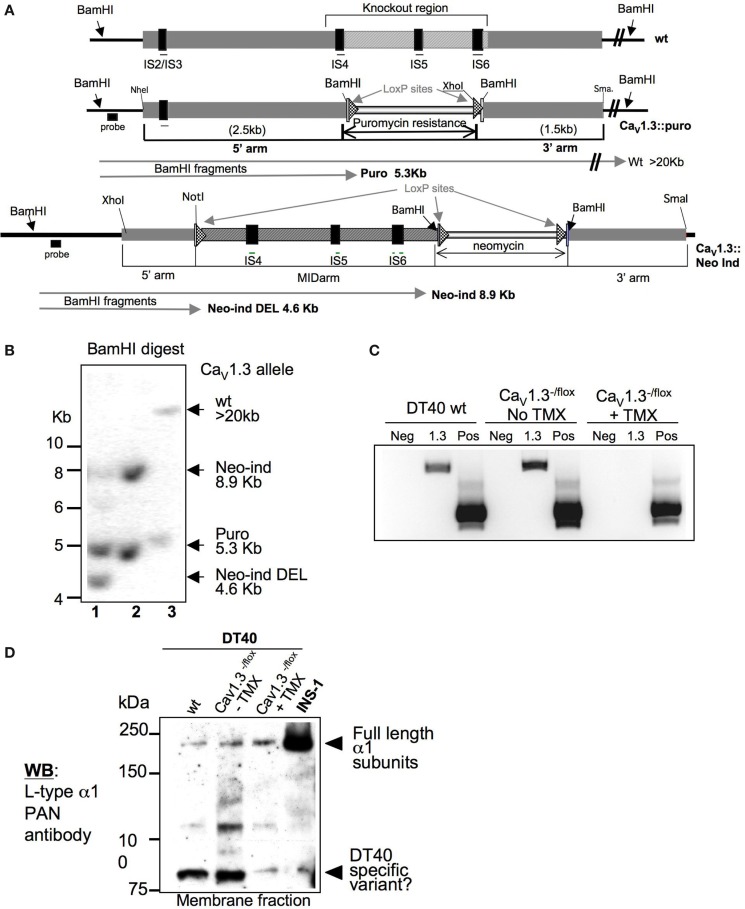
**Targeting strategy of the Ca_V_1.3 encoding gene *cacna1d*, and validation of the tamoxifen-inducible *cacna1d* disruption in the DT40 cell line: (A) Schematic representation of the magnified genomic organization of the region containing the exons encoding TM spans IS4-S6 of the chicken Ca_V_1.3 channel that was deleted is shown**. The constitutive and inducible targeting constructs are depicted underneath. The expected *Bam*HI fragment sizes by Southern are indicated by arrows. **(B)** Representative Southern blot showing one of the Ca_V_1.3^±^ clones and of the Ca_V_1.3^flox/−^ clones before (lane 1) and after tamoxifen (lane 2)-induced deletion of the *cacna1d* region of interest in comparison to a *cacna1d*^±^ clone (lane 3). **(C)** RT-PCR of wild-type DT40 cells and of one Ca_V_1.3^flox/−^ clone before and after tamoxifen (TMX)-induced deletion of the *cacna1d* region of interest using *cacna1d*-specific primers. The unrelated chicken *nudT9* gene was used as a positive control (Pos) to verify the quality of the cDNA. Neg control is RNA only. **(D)** Western blot of membrane fraction lysates of wild-type DT40s and excitable INS-1 rat pancreatic cells in comparison to one Ca_V_1.3^flox/−^ clone before and after tamoxifen-induced *cacna1d* deletion.

The results of the gene-expression and pharmacological studies in DT40s presented above confirmed the presence and potential functional relevance of L-type channels in this B-cell line. We therefore decided to utilize the high genomic plasticity of DT40 cells to generate DT40 lines in which genes encoding L-type channels are disrupted, beginning with Ca_V_1.3 α1. To this aim, we chose a targeting strategy that resulted in the deletion of exons encoding several TM regions, in particular S4, S5, and S6 that are including the putative pore-forming loop between S5 and S6 (see also Figure 1). We opted to delete the exons encoding S4–S6 of the chicken *cacna1d* gene within the first six TM group (IS4–S6). We designed a “conventional” targeting construct allowing for the exchange of the Ca_V_1.3 region of interest against a “recyclable” puromycin resistance cassette (flanked by loxP sites, generous gift from Dr. Jean-Marie Buerstedde). DT40 cells stably expressing a tamoxifen (TX)-inducible version of the Cre recombinase [MerCreMer, kindly provided by Dr. Michael Reth (61)] for potential inducible Cre-mediated genomic deletions were transfected with the targeting construct depicted in Figure 4A. We identified by Southern blot (Figure 4B) multiple DT40 clones with targeted integration of the puromycin resistance gene into the gene encoding Ca_V_1.3 with an efficiency of over 25% (5 from 19 analyzed clones). We then tried to target the second allele using a different drug resistance cassette (against histidinol), and unexpectedly did not obtain any double-targeted DT40 clones, despite screening over 50 independent cell clones, a number that in our experience is largely sufficient to isolate the desired doubly targeted mutant cells. We thus concluded that the deletion of Ca_V_1.3 is deleterious to DT40 cells, and designed an alternative targeting construct allowing for the inducible deletion of the same IS4–S6 encoding genomic region using the flox/Cre recombinase system (Figure 4A, bottom construct). Using this strategy, we were able to obtain TX-inducible deletion of the targeted Ca_V_1.3 genomic region. We determined by RT-PCR that following TX-treatment and excision of the floxed *cacna1d* region, the complete Ca_V_1.3 transcript appears to be missing in these DT40 cells (Figure 4C). The analysis of total Ca_V_1 proteins detected with the PAN antibody by immunoblotting showed that the high-molecular band (>200 kDa), which putatively represents all full-length Ca_V_1 channels in DT40s, is still present after deletion of Ca_V_1.3. This result might have been expected since our gene expression studies have demonstrated that Ca_V_1.2 and Ca_V_1.4 are also present in DT40s (Figure 2A). Noticeably, a smaller (~90 kDa) band seems to disappear in Ca_V_1.3^−/−^ DT40 cells (Figure 4D). Although we need to confirm this result by cloning the Ca_V_1.3 version(s) expressed in DT40 cells in the future, a possible interpretation is that Ca_V_1.3 is not (solely) expressed as a full-length ion channel in DT40s. This is consistent with findings described above that both in B- and T-lymphocytes, L-type variants shorter than their excitable counterparts can be identified. The characterization of immune variants of L-type channels will be essential to elucidate the role of these proteins in this context. As discussed in section *topology, nomenclature, and regulation of voltage-gated Ca^2+^ channels* of this article, immune splice variants are known which lack some of the TM spans containing the voltage sensor, consistent with the observation that depolarization does not appear to activate Ca^2+^-signals in immunocytes. It is also conceivable that some of the truncated Ca_V_ versions might actually not function as ion transport pathways, and instead fulfill other cellular roles, as illustrated by studies mentioned previously showing the ability of C-terminal portions of Ca_V_ proteins to translocate into the nucleus to act as transcriptional regulators (26, 27).

**Figure 5:**
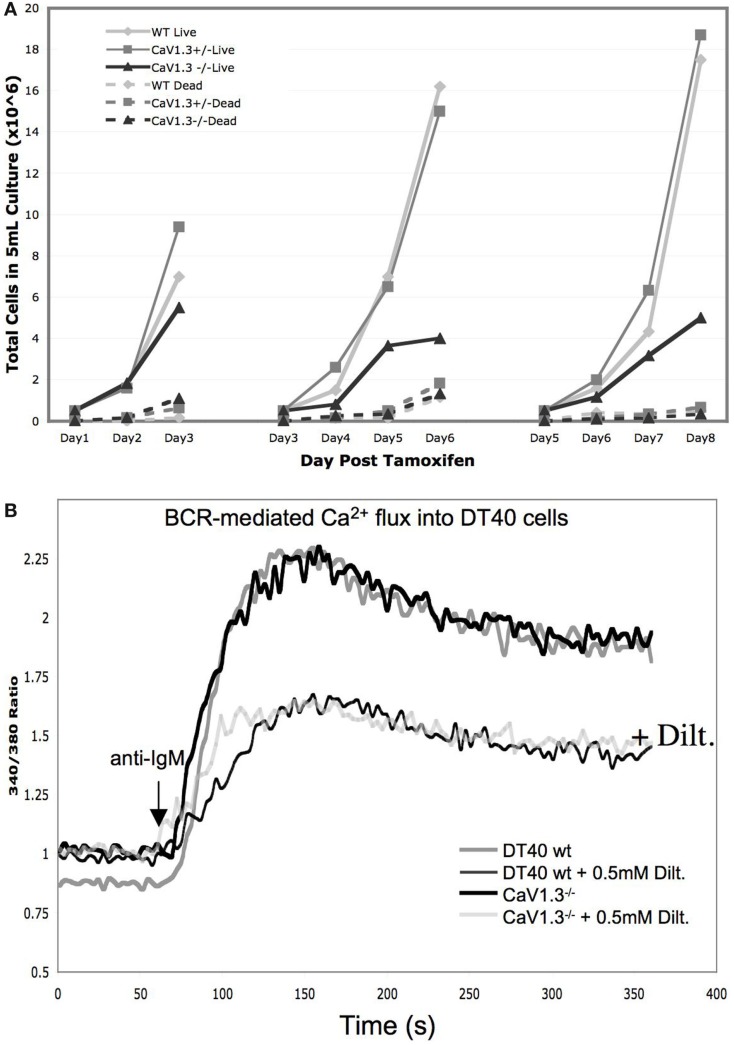
**Phenotypic characterization of the Ca_V_1.3-deficient DT40 cells: (A) Growth curves of DT40 wild-type cells (light gray) in comparison to the Ca_V_1.3^−/flox^ cells before (dark gray) and after deletion (black) of the floxed region following tamoxifen treatment**. Dashed lines represent the corresponding numbers of dead cells as determined by trypan blue in the same cultures. Cells were split back every 3 days to 0.5 × 10^6^/ml. **(B)** Ca^2+^-transients in wt vs. Ca_V_1.3-deficient DT40 cells following BCR-activation with or without L-type channel blocker diltiazem.

We found that following deletion of Ca_V_1.3, DT40 cells show a marked decrease in growth rate (Figure 5A), which is potentially causal to our failure to obtain DT40 clones constitutively deficient in Ca_V_1.3 using a conventional targeting strategy. Based on simple counts of dead cell bodies, it does not appear that cell death is substantially increased in the Ca_V_1.3^−/−^ DT40s, perhaps pointing at a defect in cell proliferation, rather than a deregulation of apoptotic, necrotic, or cell survival pathways (Figure 5A, dashed lines), although this will need to be more carefully investigated in the future. We analyzed the effect of the Ca_V_1.3 deletion on the Ca^2+^-response triggered by anti-IgM and thapsigargin treatment, and did not observe any change in the extent or shape of the Ca^2+^-responses under these conditions, nor did we see a difference in the effect of diltiazem on the Ca^2+^-increase (Figure 5B). Although speculative, this result might suggest that Ca_V_1.3′s biological effect is not mediated by Ca^2+^ influx, which might be further corroborated by the observation discussed above that a protein substantially smaller than full-length Ca_V_1.3 is seeing as missing in the Ca_V_1.3^−/−^ cells. Alternatively, it might be that Ca_V_1.3 is not activated downstream of BCR ligation, and that we need to test other triggers, for example, through GPCRs. We have indications that Ca_V_1.2 deletion in DT40s also requires an inducible strategy, suggesting that Ca_V_1.2 and Ca_V_1.3 fulfill non-redundant roles in DT40s. Ultimately, for a more complete picture of the function of these channels in DT40s to be drawn, a full set of Ca_V_1-deficient DT40 lines will need to be generated, including cell lines with combined deletions of Ca_V_1.2, Ca_V_1.3, and Ca_V_1.4. These cell lines will represent a useful set of reagents to assess the respective contribution of these different pore-forming Ca_V_ subunits in the context of BCR-signaling.

## L-Type Channels in Immune Cells of the Myeloid Lineage

Insights into the possible involvement of L-type channels in the development, homeostasis, or biological function of cells of the myleoid lineage are few. It is, however, known that L-type channel blockers such as diltiazem, which are clinically widely used to treat hypertension, also have anti-inflammatory effects (62). This could have clinical implications as pointed out in a recent study demonstrating the beneficial effect of diltiazem to prevent aneurysm formation in a mouse model through the inhibition of inflammatory cytokine production by monocytic cells (18).

In neutrophils, pharmacological inhibitors of L-type channels have been reported to reduce the Ca^2+^ response of human polymorphonuclear neutrophils (PMNs) to a neuropeptide (63), and to reduce the release of elastases and the production of ROS from these cells via diminished cytosolic Ca^2+^ mobilization and PKC activation (64). In monocytes, it was similarly reported that nifedipine dampens superoxide production and that in addition it directly contributes to reducing PKC activity (65). It has also been proposed that the anti-inflammatory effects of L-type channel blockers is reinforced by the effects of these drugs on suppressing the participation of plasminogen leading to the inhibition of macrophage emigration through tissues (66, 67). In peripheral blood-derived human dendritic cells (DCs), an early study found that apoptotic body engulfment and IL-12 production are inhibited by nifedipine (68). More recently a group proposed a model in which similar to the situation in cardiac and skeletal muscles, membrane depolarization in immature human DCs results in Ca_V_1.2 activation, which in turn triggers intracellular Ca^2+^ release via ryanodine receptor 1 (RyR1), resulting in the rapid delivery of MHC class II molecules to the plasma membrane (69). The role of RyR1 in DCs is further supported by the finding that mice carrying a gain of function mutation in RyR1 exhibit enhanced DC function (70).

Collectively, theses results suggest that beyond lymphocytes, L-type channels also play a significant role in immune cells of the myeloid lineage, which will be important to further shed light on.

## Concluding Remarks

Calcium signals in non-excitable cells such as immune cells are as diverse as the biological processes they regulate. In order to accommodate this need, immune cells rely on an equally diverse set of ion channels that unexpectedly includes molecules thought to be signature molecules of excitability, such as the L-type channels discussed in this article. Many questions remain to be addressed, such as the nature of the immune-specific L-type channel variants expressed in defined subsets of immunocytes, or the important but still elusive mechanism of activation of these immune Ca_V_1 variants since classic depolarization-mediated activation like in excitable cells do not seem to be a major factor in immune cells. In this context, it might be relevant that a voltage-gated sodium channel was recently reported to be crucial for positive selection of CD4^+^ T-lymphocytes (10), opening up the possibility that during specific biological processes, and in some distinct cell types that harbor the appropriate molecular equipment, voltage-gating of L-type channels might be an option, although this remains speculative at this time.

Despite these gaps in knowledge it seems now well established and accepted in the field that L-type channels are a force to be reckoned with in the context of immunity and inflammation. As the activity of these channels can be manipulated for therapeutic purposes, and the medical community has ample experience with drugs targeting L-type channels in the context of hypertension and cardiac conditions, there is a very real and promising potential to utilize these compounds for immunomodulatory and anti-inflammatory purposes in the future.

## Materials and Methods

### Cell culture

The DT40 cell lines were cultured in RPMI supplemented with Pen/Strep, 10% FBS, and 1% chicken serum (Sigma). DT40 wt cells stably expressing the Tet-repressor were transfected with the targeting constructs for chicken Ca_V_1.3 described under “Generation of a Ca_V_1.3-deficient DT40 B-cell line.”

### Generation of a cre-inducible Ca_V_1.3-deficient DT40 B-cell line

Chicken *cacna1d* (Ca_V_1.3) genomic DNA was obtained by screening NCBI chicken genome. The conventional targeting vectors [*cacna1d*::puromycin (puro)] were constructed by replacing the genomic fragment containing exons encoding the structurally essential TM regions IS4 to IS6 of the Ca_V_1.3 pore-forming subunit with puromycin cassettes. This cassette was flanked by 2.5 and 1.5 kb of chicken *cacna1d* genomic sequence on the 5′ and 3′ sides, respectively. The predicted DNA sequences of all constructs were verified by sequencing. The linearized targeting construct, *cacna1d*:puromycin was introduced into DT40 cells by electroporation and the cells were subsequently set under selection in serial dilutions to ensure the obtention of single clones.

Genomic DNA for Southern blot was isolated from drug-resistant DT40 cell clones, and digested with *Bam*HI. Restriction enzyme sites, probe for Southern blot analysis (solid bar) and targeted exons are indicated in Figure 4. *Bam*HI fragments detected by the probe are shown for wild-type and mutated alleles. The first allele targeting using the *cacna1d*:puro construct resulted in homologous recombination with a frequency of 20%. The targeting of the second allele using a different drug resistance (histidinol), however, failed. We therefore concluded that constitutional homozygous disruption of both *cacna1d* alleles is deleterious to DT40 cells. We thus designed and generated an inducible targeting construct allowing for the Cre-recombinase-mediated deletion of the floxed *cacana1d* region of interest (Figure 4A). The MerCreMer hybrid protein we used was kindly provided by Dr. Michael Reth (Freiburg, Germany), and allows for TX-inducible activation of the Cre-recombinase activity. The successful integration of the *cacna1d*:Neo-ind construct was verified by Southern blot, as well as the Cre-mediated excision of the deleted region following addition of TX to the media (Figure 4B).

### Expression analysis by RT-PCR

RT-PCR was performed with chicken DT40 WT RNA for the reverse transcription using a Superscript III kit ssDNA synthesis kit from Invitrogen following the manufacturer’s protocol. The PCR was performed with the Advantage pcDNA Polymerase Mix from Clontech, and the following chicken specific oligonucleotides were used:

- Ca_V_1.2: ACTTCAGATGGGCCAAAACTCTTCC
  CACCTCTTGGAGGCACAGGAGTGAAGG
- Ca_V_1.3: TACAGGAATGGCACACAGCATCGC
  CAATGAAGCACGTCCATCTTTTGGC

PCR (125 ng of single-strand DNA per reaction) was performed using standard techniques, a 2-step program for 35 cycles of 94°C for 20 s, and 60°C for 45 s. The DNA bands have been visualized onto an ethidium bromide stained 1% agarose gel with a gel documentation system (Bio-Rad). The fragments were cloned and sequenced.

### Immunoblotting

0.1–5 × 10^6^ DT40 wt or mutant cells were plated, cells lysed and proteins of the cell membrane fraction were separated by SDS/PAGE using 8% polyacrylamide gels, and transferred to a PVDF membrane. The membranes were analyzed using L-type α1 PAN antibody from Alomone Labs.

### Calcium measurements

Cytosolic [Ca^2+^] was evaluated in the indicated DT40 cell lines using the fluorescent Ca^2+^-sensitive dye fura-2. 4 × 10^6^ cells were loaded with 1 μg/ml Fura-2 (Invitrogen) for 30 min at 25°C in Ringer buffer and analyzed using a bulk assay in a spectrofluorometer (Photon Technology International) as previously described (71). L-type channel inhibitors were ordered from sigma.

## Conflict of Interest Statement

The authors declare that the research was conducted in the absence of any commercial or financial relationships that could be construed as a potential conflict of interest.
